# Developing a questionnaire to evaluate an automated audit & feedback intervention: a Rand-modified Delphi method

**DOI:** 10.1186/s12913-024-10915-2

**Published:** 2024-04-05

**Authors:** Ine Van den Wyngaert, Gijs Van Pottelbergh, Kristien Coteur, Bert Vaes, Steve Van den Bulck

**Affiliations:** 1 Academic Centre for General Practice, Department of Public Health and Primary Care, University of Leuven, Leuven, Belgium; 2 Research Group Healthcare and Ethics, Faculty of Medicine and Life Sciences, UHasselt, Diepenbeek, Belgium

**Keywords:** Medical audit, Quality of health care, Audit and feedback, Process evaluation, Delphi method, Questionnaire

## Abstract

**Background:**

Audit and feedback (A&F) is a widely used implementation strategy to evaluate and improve medical practice. The optimal design of an A&F system is uncertain and structured process evaluations are currently lacking. This study aimed to develop and validate a questionnaire to evaluate the use of automated A&F systems.

**Methods:**

Based on the Clinical Performance Feedback Intervention Theory (CP-FIT) and the REFLECT-52 (REassessing audit & Feedback interventions: a tooL for Evaluating Compliance with suggested besT practices) evaluation tool a questionnaire was designed for the purpose of evaluating automated A&F systems. A Rand-modified Delphi method was used to develop the process evaluation and obtain validation. Fourteen experts from different domains in primary care consented to participate and individually scored the questions on a 9-point Likert scale. Afterwards, the questions were discussed in a consensus meeting. After approval, the final questionnaire was compiled.

**Results:**

A 34-question questionnaire composed of 57 items was developed and presented to the expert panel. The consensus meeting resulted in a selection of 31 questions, subdivided into 43 items. A final list of 30 questions consisting of 42 items was obtained.

**Conclusion:**

A questionnaire consisting of 30 questions was drawn up for the assessment and improvement of automated A&F systems, based on CP-FIT and REFLECT-52 theory and approved by experts. Next steps will be piloting and implementation of the questionnaire.

**Supplementary Information:**

The online version contains supplementary material available at 10.1186/s12913-024-10915-2.

## Background

Audit and feedback (A&F) is a widely used implementation strategy, delivered to health care providers to evaluate and improve medical practice. In 2003, Jamtvedt et al. defined A&F as ‘any summary of clinical performance of health care over a specified period of time, given in a written, electronic or verbal format’ [1].

Several studies have shown the effectiveness of A&F systems in primary care [1–5]. Even though the effect of A&F seems to be variable and depends on how the feedback is given [4]. When A&F is not optimally delivered, the performance of the care provider, and thus the care received by patients, can be negatively affected [4]. However, the optimal design of a digital and automated A&F system (the health care provider will automatically receive a digital feedback report) is still uncertain, but several components of such an optimal system are suggested by earlier research [3, 4]. In addition, there is no gold standard available for implementation of an A&F intervention [3]. In primary care, digital and automated A&F based on data stored in electronic health records (EHRs) is currently used [6]. Automated A&F systems would have the potential to save time and to drive continuous quality improvement, although there are still some challenges that need to be solved first [7]. Automation could also increase the effectiveness of feedback, since even participants with a lack of time or skills can easily receive feedback [8]. In addition, an automated A&F system could contribute to more patient-centred care, which various studies describe as a promising framework to meet the challenges in our complex health care system [9–11]. An automated A&F system enables health care providers to detect gaps in the care for specific patient groups, after which care tailored to those patients can be achieved [12, 13].

To improve the effectiveness of an automated A&F system and to avoid waste of research and economic sources, repeated optimization of the A&F system is necessary [14]. Since an automated A&F system is a complex intervention, an essential part of designing, testing and adjusting, is a process evaluation. This can be used to assess the reliability and quality of the implementation. In addition, a process evaluation can clarify causal mechanisms and identify contextual factors associated with variation in outcomes [15–17]. Observational research suggested that general practitioners (GP) have preferences regarding the types of feedback they would like to receive [18]. Including their assessment of the automated A&F system during the process evaluation is therefore certainly useful. Different theories have been developed to help explain what factors influence feedback success, such as Control Theory, Goal setting Theory and Feedback Intervention Theory [19–21]. In 2019, the Clinical Performance Feedback Intervention Theory (CP-FIT) was described, which is the most comprehensive theory to explain the effectiveness of A&F [8]. However, a practical and validated application of these theories to allow automated A&F systems to be assessed by the care provider, is currently lacking.

The aim of this study was to develop a questionnaire to evaluate the use of automated A&F systems and to achieve content validity. Mapping the perceived importance and barriers of health care providers when working with automated A&F systems, could improve the quality and implementation of future automated A&F interventions.

## Methods

### Study design

A Rand-modified Delphi method was used for the development of the questionnaire [22]. This method is used to obtain the consensus among experts, based on the theoretical frameworks available in literature [8, 23]. It includes 5 stages: (1) Drafting questions based on scientific literature (CP-FIT and REFLECT-52 evaluation tool) (2) Individual assessment of the questions by an expert panel, analysis of these results and drawing up a personal feedback report (questionnaire round) (3) A consensus round consisting of a panel discussion with the experts based on the feedback report (4) Final assessment of the questionnaire by the expert panel (final evaluation) (5) Processing these assessments in the final questionnaire (final questionnaire).

### Study population

The expert panel initially consisted of 14 members with Belgian nationality, 9 men and 5 women. The selection of experts was based on the work of Concannon and Parker et al. [24, 25]. The panel was composed of four principal investigators, four providers and six policy makers (See Additional file 1). Among them were seven medical doctors (six general practitioners, one paediatrician), three pharmacists, one nurse, one psychologist and 2 researchers. After the questionnaire round, one of the pharmacists decided not to participate further. Among the panel members were 2 GPs with experience in developing A&F systems.

### Drafting questions

We conducted a literature research on the possible theoretical frameworks. Based on the CP-FIT and the REFLECT-52 evaluation tool a questionnaire was designed for the purpose of evaluating the use of A&F systems [8, 23]. CP-FIT explains how effective feedback works in a cycle of sequential processes, starting with goal setting, followed by data collection and analysis, giving feedback, reception, comprehension and acceptance of this by the recipient, a planned behavioral response based on the feedback and improvement of clinical performance. The cycle then repeats. Feedback interventions become less effective if any individual step in this process cycle fails. CP-FIT describes 42 hypotheses that influence the feedback cycle and thus the feedback’s success, operating via three mechanisms: the feedback method used, the feedback recipients and the context [8]. The REFLECT-52 tool consists of 52 items that can serve as a basis to assess the conformity to best practices in existing A&F interventions. It focuses on the nature of the desired action, the nature of the data available for feedback, the feedback display and delivering the feedback intervention [23].

### Data collection and analysis

#### Questionnaire round

##### Online assessment

**Fig. 1:**
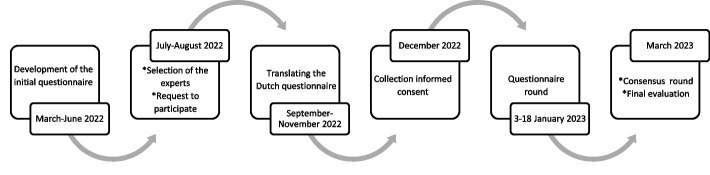
Timeline of the research

The expert panel was asked to assess the prepared questions online for relevance for inclusion in the questionnaire (See Additional file 2). They were asked to answer them within 2 weeks (See Fig. 1). Each question was rated using a Likert scale, with 1 being the lowest score (not very relevant question) and 9 being the highest score (highly relevant question). If a panel member could not rate the question, an option 'not evaluable' was added. Comments and suggestions for adjustments could be noted per question. After each part, panelists were asked to rank the questions in a top 3 or top 5 (prioritization). At the end of the questionnaire, participants had the opportunity to formulate suggestions for new questions.

##### Analysis

After the online survey, a personal feedback form was drawn up for each expert. The scores on the 9-point Likert scale awarded by all participants, the median and top-percentage, as well as the participant’s own score, argumentation and suggestions were reflected in the feedback report. For each question, it was indicated whether it was considered suitable for inclusion in the questionnaire, whether its relevance was under discussion or was insufficient. The median Likert scale scores and prioritization were used to determine the degree of agreement between the members of the expert panel. Based on preselection and consensus, an initial assessment was formulated for each question and this was also reflected in the personal feedback form.

##### Median Likert scale scores

The median of all panelists' scores for each recommendation, ranging from 1 to 9.

##### Prioritization

Prioritization was a percentage based on the score of the question in a top-5 or top-3. The first ranked question received 5 points, the second 4 points, etc. If a question was not included in the top-5 or top-3 listing, it received 0 points. Individual prioritization points were then added up and divided by the maximal possible points of the question. For example, if 10 panelists ranked a recommendation first and 4 did not mention it in their top-5 score, the top percentage was 50/70 (14 x 5 = 70) or 71.4%.

##### Preselection and consensus

Criteria for preselection and consensus are shown in Table 1.

**Table 1:** Preselection and consensus criteria

**Preselection**	Median Likert score ≥ 7 and top percentage ≥ 20%	Selection
	Median Likert score ≥ 7 and the top percentage between 1-20%	Discussion
	Median Likert score < 7 and top percentage ≥20%	Discussion
	Other	No selection
**Consensus**	≥70% of median Likert scores in highest tertile	Consensus
	≥ 30% of median Likert scores in highest tertile and ≥ 30% in lowest tertile	Discussion
	Other	No consensus

##### Classification of questions

Based on the preselection and consensus, the questions were marked as ‘selected’, ‘up for discussion’, or ‘not selected’. The questions selected in this first round were marked in green. The questions that were under discussion were colored orange and those who were not selected, were marked in red.

### Consensus round

The questions and the feedback report were reviewed during an online panel discussion. The questions selected in the questionnaire round (green color) were briefly discussed for inclusion, those who were not selected (red color) for exclusion. Those under discussion (orange color) were reviewed more extensively for inclusion or exclusion.

### Final evaluation

After the consensus round, the modified questionnaire was submitted to the expert panel by e-mail for their final approval.

### Final questionnaire

After processing the last comments, the final questionnaire was drawn up.

## Results

### Drafting questions

**Fig. 2:**
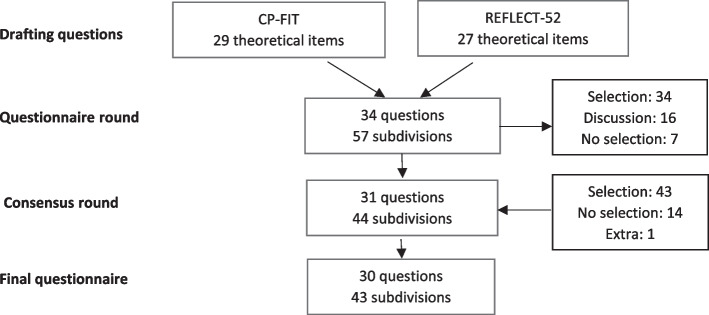
Different phases of development of the questionnaire

Out of the 42 items proposed by the CP-FIT theory, 29 items were selected as the basis for our questionnaire. This was supplemented with 27 items suggested by the REFLECT-52. Some of the selected items occurred in both theories (See Fig. 2 and Additional file 2). After selection, a questionnaire of 34 questions according to the rules of mixed-method research [26, 27] was composed in Dutch. The questions covered different topics of the automated A&F system such as care giver and practice data, the purpose of the questionnaire, collection of the data, the feedback given and co-interventions. The initial survey was discussed by three researchers (IVDW, SVDB, KC) until agreement was reached that the questions adequately represented the key components of each selected theory based item. The survey was refined in terms of content and linguistics and translated into French and English by sworn translators. The response categories (I totally agree/ I rather agree/ I am neutral/ I rather disagree/ I totally disagree) were chosen to facilitate quantitative summaries. The remaining items used a combination of open questions and descriptive or numerical response categories to provide further details on the A&F intervention. Before the questionnaire was presented to the panel of experts, the questionnaire was completed by four GPs to detect technical errors.

### Questionnaire round

The response rate when assessing the 34-question questionnaire was 100%. Some questions were divided into several sub-questions and statements, resulting in a total of 57 items to be assessed. Based on the preselection and consensus results, 34 out of 57 items were selected, 16 needed further discussion and 7 were not withheld (See Fig. 2).

### Consensus round

Ten panellists attended the consensus meeting. In this round, 31 out of the 34 preselected items were kept. Ten items from the group under discussion (see Additional file 2, question 2, 7, 8, 9, 11, 19, 23.7, 27.2, 27.5, 32) and two of the ‘not selected’ questions (see Additional file 2, question 3 and 31) were also selected. The panel found the content of the questions valuable, but the questions were not well formulated. During the consensus meeting, the manner of posing the questions was considered, after which they could be included in the questionnaire. One completely new question was added regarding the payment system used (See Table 2, question 6). Question 14.2, 14.4, 14.5, 23.5, 23.8, 24.1, 27.6, 27.7, 27.8, 28 and 33 (see Additional file 2) were not selected by the expert panel, although some were selected during questionnaire round (question 27.9 and 28). The consensus round resulted in a selection of 44 items, distributed over 31 questions.

**Table 2:** Final questionnaire

**Number**	**Description**
**Part 1**	**This section questions caregiver and practice data**
**Question 1**	*What role do you assume as a caregiver?*
	• General Practitioner (GP)
	• Trainee GP
	• Specialist
	• Practice assistant
	• Nurse
	• Home nursing
	• Pharmacist
	• Physiotherapist
	• Dentist
	• Other
	Specify: (open)
**Question 2**	*How long have you been professionally active as a healthcare provider?*
	• <5 years
	• 5-10 years
	• 11-20 years
	• 21-30 years
	• >30 years
**Question 3**	*What is your gender?*
	• Man
	• Woman
	• X
	• I don’t want to say
**Question 4**	*Where is your practice located?*
	• Postcode: (structured list)
**Question 5**	*What type of practice do you mainly work in?*
	• Solo practice/ pharmacy (1 health care provider)
	• Duo practice/ pharmacy (2 health care providers, the same discipline)
	• Monodisciplinary group practice/ team (3 or more health care providers, the same discipline)
	• Multidisciplinary group practice/ team (3 or more health care providers, different disciplines)
	• Community health center (3 or more health care providers, an organized collaboration between at least general practitioner medicine, a paramedical discipline and a discipline of social work, flat-rate payment system)
	• Hospital
	• Other: (open)
**Question 6**	*What payment system do you work with?*
	• Flat-rate payment system
	• Performance-based system
**Question 7**	*Does your practice or team employ a practice assistant and/or administrative assistant?*
	• Yes
	• No
**Question 8**	*Does your practice currently employ a trainee general practitioner?*
	• Yes
	• No
**Question 9**	*How many in-person patient contacts (consultation and home visits) do you have per health care provider on an average weekday?*
	• ≤10
	• 11-20
	• 21-30
	• 31-40
	• >40
**Question 10**	*Did you participate at least once in the Audit?*
	• Yes
	• No
**Question 11**	*Why did you not participate in the Audit? (open)*
**Part 2.1**	**Purpose of the survey**
**Question 12**	*Please indicate the extent to which you agree with the following statements:*
**Question 12.1**	The purpose of the Audit is clear to me.
**Question 12.2**	I find the purpose of the Audit relevant to the work I do today.
**Question 12.3**	I find that using the Audit positively affects my current way of working.
**Part 2.2**	**Collection of data through the EMD**
**Question 13**	*How satisfied are you with the way the data was collected? (Completing and submitting the e-form in the EHR)*
	• Very satisfied
	• Rather satisfied
	• Neutral
	• Rather dissatisfied
	• Very dissatisfied
**Question 14**	*How could we improve the way we collect data?* (open)
**Question 15**	*Indicate the extent to which you agree with the following statement: I feel that the data and results retrieved are an accurate representation of my practice.*
	• Totally agree
	• Rather agree
	• Neutral
	• Rather disagree
	• Totally disagree
**Question 16**	*How satisfied are you with the frequency of data retrieval?*
	• Very satisfied
	• Rather satisfied
	• Neutral
	• Rather dissatisfied
	• Very dissatisfied
	• Totally disagree
**Question 17**	*How frequently would you like to participate in the Audit?*
	• Biweekly
	• Monthly
	• Quarterly
	• Semi-annually
	• Other: open
**Question 18**	*Indicate the extent to which you agree with the following statement: I find participation in the Audit (collecting data and sending the eForm) time consuming.*
	• Totally agree
	• Rather agree
	• Neutral
	• Rather disagree
	• Totally disagree
**Part 2.3**	**Get feedback**
**Question 19**	*How frequently did you use the feedback?*
	• Several times a week
	• Once a week
	• Several times a month
	• Once a month
	• Several times every six months
	• Once every six months
	• Never
	• Other: (open)
**Question 20**	*Why did you never look at the feedback?* (open)
**Question 21**	*Please indicate the extent to which you are satisfied:*
**Question 21.1**	How satisfied are you about how feedback is given (in general)?
**Question 21.2**	How satisfied are you about the frequency of the feedback?
**Question 21.3**	How satisfied are you about getting feedback per practice, as opposed to being able to get feedback per individual physician?
**Question 21.4**	How satisfied are you about the way the performance level of the practice is displayed by means of tables and graphs?
**Question 21.5**	How satisfied are you about the content of the feedback?
**Question 21.6**	How satisfied are you about the possibility to compare performance level of the practice with others (province/ primary care area/...) and the number of levels available for comparison? (benchmarking)
**Question 22**	*Please indicate the extent to which you agree with the following statements:*
**Question 22.1**	I find the feedback given relevant to achieving a better level of performance.
**Question 22.2**	I find it useful that the current level of performance is shown in relation to the previous level of performance (longitudinal view of performance level).
**Question 22.3**	I find the way a feedback report can be viewed user-friendly.
**Question 23**	*Did you set up any type of quality improvement project in your practice based on the feedback provided?*
	• Yes
	• No
**Question 24**	*Why did you/ didn’t you set up a quality improvement project? (open)*
**Question 25**	*Please indicate the extent to which you agree with the following statements:*
**Question 25.1**	I find the effort I have to put into reviewing the feedback negligible.
**Question 25.2**	I am satisfied with the amount of feedback given.
**Question 25.3**	The feedback should contain written advice that aims to improve my performance level and is easy to implement.
**Question 25.4**	If I received the feedback directly in my EMD (push system), I would use it more frequently.
**Question 25.5**	The feedback provided aims to support me in improving the performance level of my practice.
**Part 2.4**	**Co-interventions**
**Question 26**	*Please indicate the extent to which you agree with the following statement: A discussion with other GPs regarding the feedback received seems a useful addition in order to arrive at changes in my medical practice.*
	• Totally agree
	• Rather agree
	• Neutral
	• Rather disagree
	• Totally disagree
**Question 27**	*How satisfied are you regarding the support on using the Audit and getting feedback (webinar, film clip)?*
	• Very satisfied
	• Rather satisfied
	• Neutral
	• Rather dissatisfied
	• Very dissatisfied
	• I haven’t looked into it
**Question 28**	*How satisfied are you with the way questions and problems related to the audit were resolved?*
	• Very satisfied
	• Rather satisfied
	• Neutral
	• Rather dissatisfied
	• Very dissatisfied
	• I did not contact the helpdesk
**Question 29**	*Indicate the extent to which you agree with the following statement: The reminder helped me participate in the Audit.*
	• Totally agree
	• Rather agree
	• Neutral
	• Rather disagree
	• Totally disagree
**Question 30**	Non-mandatory question: *Is there anything else you would like to mention? (*open)

### Final evaluation

A list of 31 questions was sent to the panel for final review. The researchers asked the panel to exclude one question, because of the subjective nature of this question (See Additional file 2, question 11).

### Final questionnaire

A final list of 30 questions (43 items) was obtained (See Table 2). Main topics include caregiver and practice data, purpose of the survey, collection of the data, getting feedback and co-interventions.

## Discussion

This study used a Rand modified Delphi method to develop a process evaluation questionnaire for automated A&F interventions. Based on the CP-FIT and REFLECT-52 evaluation tool, a questionnaire with 34 questions (57 items) was designed to evaluate and improve automated A&F systems [8, 23]. After a quantitative and qualitative review of the questionnaire by a 14-person expert panel, 30 questions (43 items) remained. Covering different aspects of the A&F system, namely care giver and practice data, the purpose of the questionnaire, collection of the data, the feedback given and co-interventions, this questionnaire deemed suitable for the assessment of automated and feedback systems by their users.

In 2013, Colquhoun and colleagues compared 18 different theories that can be used in the design and evaluation of A&F systems [12]. More recently, three theories have gained more popularity, namely Control Theory, Goal setting Theory and Feedback Intervention Theory [19–21]. However, these theories describe only part of the feedback process and they lack some factors specific to health care improvement like team based change, context and intervention implementation. Since CP-FIT theory and REFLECT-52 evaluation tool contain these aspects related to health care, we used them as a basis for the first questionnaire [8, 23].

Although the first part of the questionnaire on practice and caregiver data does not directly query the automated A&F system, it is quite relevant. After all, we know that the effect of A&F depends on the recipient and does not have the same impact in different professional categories [28]. During the consensus meeting, question 1 (see Table 2) regarding the role of the healthcare provider was therefore extended to other disciplines in primary care. Some studies found that the effect of an A&F intervention was strengthened when senior physicians were brought into an audit process, initially aimed at junior colleagues [28, 29]. The expert panel decided to adjust question 2 (see Table 2) so that the number of years of professional activity was asked rather than age, as they considered this to be more relevant. Question 3 (see Table 2) regarding gender was not selected during the questionnaire round. The way the question was asked and the response options, led to discussion within the panel. A recently published scoping review states that using dichotomous response options of female and male are insufficient today, but the perfect set of options does not exist [30]. The Belgian institute for the equality of women and men advises adding an 'other' category, as well as an option ‘not to answer the question’ [31]. The expert panel chose to follow this latter advise.

Practice setting also plays a role in A&F, where CP-FIT assumes that teams and organisations have more capacity to process feedback [8, 32]. Damschroder et al. mentioned that the available resources influence the implementation process, whereby ‘time’ contributes to a positive implementation climate for an intervention [33]. In addition, people who have significant other responsibilities (e.g. healthcare providers with a crowded agenda) should be less able to handle feedback [8]. Therefore, the questions about practice type and workload (question 5, 7 and 9, see Table 2) were included and an extra question was added about the payment system (question 6, see Table 2).

The initial questionnaire assessed the participant’s digital literacy through the statement ‘I consider myself competent in using a computer’, based on the finding that people with greater clinical and technical skills, are more likely to successfully handle feedback. (See Tdditional file 2, question 11) [8, 34] After consensus round, this question was changed to ‘use of the Electronic Health Record’, but was subsequently deleted at the request of the researchers in the final evaluation due to the subjective nature of the question.

The questions about the purpose of the feedback, data collection and obtaining feedback were almost all retained in the final questionnaire. Some questions (question 14.2, 14.4, 14.5, 23.5, 23.8, 27.6, 27.7, 27.8, see Additional file 2) were deleted by the panel because their content was too similar to another question. Question 27.9 and 28 were not retained since question 25 (see Additional file 2) was converted into asking about setting up a quality improvement project (question 23, see Table 2) and thus covered the same content as questions 27.9 and 28. Both CP-FIT and REFLECT-52 state that displaying patient data used to assess the clinical performance of the health professional, facilitates the feedback mechanism by enabling recipients to understand how suboptimal care arose [8, 23]. However, the question on individual patient data (question 24.1, see Additional file 2) was excluded by the panel, as this is currently not possible in the Belgian feedback systems. An intervention is also more effective when the participant is supported (in obtaining feedback) and solving problems [8, 35]. However, the question on problem solving (question 28, see Table 2) was initially not retained during the questionnaire round. An adjustment of the question eventually led to inclusion in the list. Previous research found that a financial reward may negatively impact feedback success by counteracting the recipient’s motivation an sense of professionalism [34]. Yet this question (question 33, see Additional file 2) was not retained by the panel since as it was believed that it would not be answered fairly.

As mentioned in the introduction, an automated A&F system can play an important role in primary care. A&F acts as a ‘learning health care system’, in which data from the Electronic Health Record are quickly converted into feedback, changes in practice and care for the patient [36]. A&F also offers the opportunity to improve behavior and care at patient level, which forms the base for patient-centered care [8]. A&F can fence in population management where a specific group of patients is selected (e.g. diabetics), the care for their chronic disease is evaluated and feedback provided to the health care provided about the blind spots in care. On the basis of the results, the health care provider can give extra attention to individual patients and discuss an individualized support plan, based on the needs of the patient. Further research on other possible patient roles in automated A&F systems (active participation in feedback and service improvement) is needed, looking beyond current ways of involvement [37].

### Strengths and limitations

There are some strengths and limitations to note. The questionnaire was based on substantiated theory and the content was validated by an expert panel, which is certainly a major strength of this study. The expert panel consisted of professionals from different disciplines with multiple and different roles in primary and secondary care (see Additional file 1), so that a generic questionnaire for health care was created which can be used in both primary and secondary care One of the limitations concerns the absence of patients in the expert panel. This was deliberately considered and in the end it was decided not to allow them to participate, as they are not users of automated A&F systems. Finally, the final questionnaire has not been piloted in practice. To test the questionnaire, it will be offered to participants of A&F systems. In a next phase, it will, for example, be integrated into the Belgian EHR.

## Conclusion

A process evaluation in the form of a questionnaire consisting of 30 questions was designed, based on the CP-FIT and REFLECT-52 tool and achieved content validity by experts. This generic questionnaire can be used for the assessment and improvement of automated A&F systems in different healthcare settings.

### Supplementary Information

**Supplementary Material 1.** 

**Supplementary Material 2.** 

## Data Availability

All data generated or analysed during this study are included in this published article [and its supplementary information files].

## Abbreviations

A&F: Audit and Feedback
CP-FIT: Clinical Performance Feedback Intervention Theory
REFLECT-52: REassessing audit & Feedback interventions: a tooL for Evaluating Compliance with suggested besT practices
GP: General practitioner
EHR: Electronic Health Record

## References

[1] Jamtvedt G, Young JM, Kristoffersen DT, O'Brien MA, Oxman AD. Audit and feedback: effects on professional practice and health care outcomes. Cochrane Database Syst Rev. 2006(2):1–105. 10.1002/14651858.CD000259.pub2 16625533

[2] Glenngard AH, Anell A. The impact of audit and feedback to support change behaviour in healthcare organisations - a cross-sectional qualitative study of primary care centre managers. BMC Health Serv Res. 2021;21(1):663. 10.1186/s12913-021-06645-4 PMC8258937 34229678

[3] Flottorp SA, Jamtvedt G, Gibis B, McKee M. Using audit and feedback to health professionals to improve the quality and safety of health care. World Health Organization, on behalf of the Eur Observ Health Syst Policies. 2010:1–54.

[4] Ivers N, Jamtvedt G, Flottorp S, Young JM, Odgaard-Jensen J, French SD, et al. Audit and feedback: effects on professional practice and healthcare outcomes. Cochrane Database Syst Rev. 2012(6):1–219. 10.1002/14651858.CD000259.pub3 PMC11338587 22696318

[5] Van den Bulck S, Spitaels D, Vaes B, Goderis G, Hermens R, Vankrunkelsven P (2020). The effect of electronic audits and feedback in primary care and factors that contribute to their effectiveness: a systematic review. Int J Qual Health Care.

[6] Gulliford MC, Prevost AT, Charlton J, Juszczyk D, Soames J, McDermott L, et al. Effectiveness and safety of electronically delivered prescribing feedback and decision support on antibiotic use for respiratory illness in primary care: REDUCE cluster randomised trial. BMJ-Br Med J. 2019;364:I236. 10.1136/bmj.l236 PMC6371944 30755451

[7] Roth CP, Lim Y-W, Pevnick JM, Asch SM, McGlynn EA (2009). The challenge of measuring quality of care from the electronic health record. Am J Med Qual.

[8] Brown B, Gude WT, Blakeman T, van der Veer SN, Ivers N, Francis JJ, et al. Clinical Performance Feedback Intervention Theory (CP-FIT): a new theory for designing, implementing, and evaluating feedback in health care based on a systematic review and meta-synthesis of qualitative research. Implement Sci. 2019;14(1):40. 10.1186/s13012-019-0883-5 PMC6486695 31027495

[9] Havas K, Douglas C, Bonner A. Meeting patients where they are: improving outcomes in early chronic kidney disease with tailored self-management support (the CKD-SMS study). BMC Nephrol. 2018;19(1):279. 10.1186/s12882-018-1075-2 PMC6195997 30342487

[10] Lee MC, Wu SFV, Hsieh NC, Tsai JM (2016). Self-management programs on eGFR, depression, and quality of life among patients with chronic kidney disease: a meta-analysis. Asian Nurs Res.

[11] Stenberg U, Haaland-Overby M, Fredriksen K, Westermann KF, Kvisvik T (2016). A scoping review of the literature on benefits and challenges of participating in patient education programs aimed at promoting self-management for people living with chronic illness. Patient Educ Counsel.

[12] Colquhoun HL, Brehaut JC, Sales A, Ivers N, Grimshaw J, Michie S, et al. A systematic review of the use of theory in randomized controlled trials of audit and feedback. Implement Sci. 2013;8:66. 10.1186/1748-5908-8-66 PMC3702512 23759034

[13] Anders A. Performance management and audit & feedback to support learning and innovation – theoretical review and implications for Swedish primary care. Papers in innovation studies paper no. 2019/11. Lund: Centre for Innovation, Research and Competence in the Learning Economy (CIRC LE) Lund University. 2019;11:1–20.

[14] Ivers NM, Grimshaw JM (2016). Reducing research waste with implementation laboratories. Lancet.

[15] Craig P, Dieppe P, Macintyre S, Michie S, Nazareth I, Petticrew M. Developing and evaluating complex interventions: the new Medical Research Council guidance. Br Med J. 2008;337(7676):a1655. 10.1136/bmj.a1655 PMC2769032 18824488

[16] Moore GF, Audrey S, Barker M, Bond L, Bonell C, Hardeman W, et al. Process evaluation of complex interventions: Medical Research Council guidance. BMJ-Br Med J. 2015;350h1258. 10.1136/bmj.h1258 PMC4366184 25791983

[17] Glidewell L, Hunter C, Ward V, McEachan RRC, Lawton R, Willis TA, et al. Explaining variable effects of an adaptable implementation package to promote evidence-based practice in primary care: a longitudinal process evaluation. Implement Sci. 2022;17(1):9. 10.1186/s13012-021-01166-4 PMC8793205 35086528

[18] Sebo P, Maisonneuve H, Fournier JP, Senn N, Haller DM. General practitioners' views and preferences about quality improvement feedback in preventive care: a cross-sectional study in Switzerland and France. Implement Sci. 2017;12(1):95. 10.1186/s13012-017-0623-7 PMC5530524 28747187

[19] Carver CS, Scheier MF (1982). Control-theory: a useful conceptual framework for personality, social, clinical and health psychology. Psychol Bull.

[20] Locke EA, Latham GP (2002). Building a practically useful theory of goal setting and task motivation - A 35-year odyssey. Am Psycholo.

[21] Kluger AN, DeNisi A (1996). The effects of feedback interventions on performance: a historical review, a meta-analysis, and a preliminary feedback intervention theory. Psychol Bull.

[22] Dalkey N, Helmer O (1963). An experimental application of the Delphi method to the use of experts. Manag Sci.

[23] Foster M, Presseau J, Podolsky E, McIntyre L, Papoulias M, Brehaut JC. How well do critical care audit and feedback interventions adhere to best practice? Development and application of the REFLECT-52 evaluation tool. Implement Sci. 2021;16(1):81. 10.1186/s13012-021-01145-9 PMC8369748 34404449

[24] Concannon TW, Grant S, Welch V, Petkovic J, Selby J, Crowe S (2019). Practical guidance for involving stakeholders in health research. J Gen Intern Med.

[25] Parker R, Tomlinson E, Concannon TW, Akl E, Petkovic J, Welch VA (2022). Factors to Consider During Identification and Invitation of Individuals in a Multi-stakeholder Research Partnership. J Gen Intern Med..

[26] Johnson RB, Onwuegbuzie AJ, Turner LA (2007). Toward a definition of mixed methods research. J Mixed Methods Res.

[27] Jansen H. De kwalitatieve survey: Methodologische identiteit en systematiek van het meest eenvoudige type kwalitatief onderzoek. Kwalon. 2005;10(3):15–34.

[28] Mugford M, Banfield P, Ohanlon M (1991). Effects of feedback of information on clinical practice - a review. Br Med J.

[29] Cialdini RB. Influence: the psychology of persuasion. Cambridge: Collins; 2007. p. 1–30.

[30] Young SK, Bond MA. A scoping review of the structuring of questions about sexual orientation and gender identity. J Commun Psychol. 2023;51(7):2592–617. 10.1002/jcop.23048 37088990

[31] Van Hove H. Gender op een inclusieve manier bevragen. Onderzoeksnota bij de enquête #YoutToo? Brussel: Instituut voor de gelijkheid van vrouwen en mannen. 2022:1–6.

[32] Axtadam P, Vanderwouden JC, Vanderdoes E (1993). Influencing behavior of physicians ordering laboratory tests - a literature study. Med Care.

[33] Damschroder LJ, Aron DC, Keith RE, Kirsh SR, Alexander JA, Lowery JC. Fostering implementation of health services research findings into practice: a consolidated framework for advancing implementation science. Implement Sci. 2009;4:50. 10.1186/1748-5908-4-50 PMC2736161 19664226

[34] Michie S, van Stralen MM, West R. The behaviour change wheel: A new method for characterising and designing behaviour change interventions. Implement Sci. 2011;6:42. 10.1186/1748-5908-6-42 PMC3096582 21513547

[35] Ammenwerth E, Iller C, Mahler C (2006). IT-adoption and the interaction of task, technology and individuals: a fit framework and a case study. BMC Med Inform Decis Mak.

[36] Jeffries M, Keers RN, Phipps DL, Williams R, Brown B, Avery AJ, et al. Developing a learning health system: Insights from a qualitative process evaluation of a pharmacist-led electronic audit and feedback intervention to improve medication safety in primary care. Plos One. 2018;13(10):e0205419. 10.1371/journal.pone.0205419 PMC6203246 30365508

[37] Foy R, Skrypak M, Alderson S, Ivers NM, McInerney B, Stoddart J, et al. Revitalising audit and feedback to improve patient care. Bmj-Br Med J. 2020;368:m213. 10.1136/bmj.m213 PMC7190377 32107249

