# Supplementary material for: Developing a questionnaire to evaluate an automated audit & feedback intervention: a Rand-modified Delphi method
Source: BMC Health Serv Res. 2024 Apr 5;24:433. doi: 10.1186/s12913-024-10915-2 (PMC10998400; doi:10.1186/s12913-024-10915-2)
Supplement: Supplementary file 1 — Supplementary Material 1. [file 12913_2024_10915_MOESM1_ESM.pdf]

Additional file 1

This additional file concerns the characteristics of the participating panelists.

Participant	Organization	Type of organization/ description	Type of healthcare provider	Concannon 2012 framework
1	Vivel	Umbrella organization	Psychologist	Policy maker
2	BeHive	Academic	Nurse	Principal investigators
3	Paqs	Care quality	Researcher	Policy maker
4	Cebam	Evidence Based Practice	Researcher Biomedical sciences	Policy maker
5	Cebam	Evidence Based Practice	General practitioner	Policy maker
6	Bapcoc	Federal Government	Pharmacist	Policy maker
7	KCE	Research	Pediatrician	Principal investigators
8	Domus Medica	Professional organization of health care providers	General practitioner	Providers
9	APB	Professional organization of health care providers	Pharmacist	Providers
10	APB	Professional organization of health care providers	Pharmacist	Providers
11	ACHG	User EHR	General practitioner	Providers
12	ACHG	Coordinator integrated care projects	General practitioner	Policy maker
13	ACHG	Research	General practitioner	Principal investigators
14	ACHG	Research	General practitioner	Principal investigators

Vlaams Instituut Voor de Eerste Lijn (Vivel) – Plateforme pour l'Amélioration continue de la Qualité des soins et de la Sécurité des patients (Paqs) - Centrum Voor Evidence-Based Medicine (Cebam) – Belgische Commissie voor de Coördinatie van het antibioticabeleid (BAPCOC) – Federaal Kenniscentrum voor de Gezondheidszorg (KCE) – Algemene Pharmaceutische Bond (APB) – Academisch Centrum voor Huisartsgeneeskunde (ACHG)
