# Supplementary material for: Developing a questionnaire to evaluate an automated audit & feedback intervention: a Rand-modified Delphi method
Source: BMC Health Serv Res. 2024 Apr 5;24:433. doi: 10.1186/s12913-024-10915-2 (PMC10998400; doi:10.1186/s12913-024-10915-2)
Supplement: Supplementary file 2 — Supplementary Material 2. [file 12913_2024_10915_MOESM2_ESM.pdf]

Additional file 2

This additional file concerns the initial questionnaire that was submitted to the expert panel in the online assessment.

Part 1 – This section questions caregiver and practice data

1) What role do you assume as a caregiver?
    a. General Practitioner (GP)
    b. Practice assistant
    c. Nurse
    d. Specialist
    e. Other
        i. Specify: (open)

- With this question, more information is obtained about the characteristics of the health professional. It is not based on any of the hypotheses of the CP-FIT or REFLECT-52 theory. However, this question does connect to the theme of ‘health professional characteristics’ from the CP-FIT theory.
- To what extent do you think the content of this question is relevant to be included in the survey? (score 1 = not very relevant, score 9 = very relevant)
    - Scale 1-9, 'do not rate' option.
    - Justify your choice: (open)
    - Suggestions for modification: (open)

2) How old are you? (open)
- With this question, more information is obtained about the characteristics of the health professional. It is not based on any of the hypotheses of the CP-FIT or REFLECT-52 theory. However, this question does connect to the theme of ‘health professional characteristics’ from the CP-FIT theory.
- To what extent do you think the content of this question is relevant to be included in the survey? (score 1 = not very relevant, score 9 = very relevant)
    - Scale 1-9, 'do not rate' option.
    - Justify your choice: (open)
    - Suggestions for modification: (open)

3) What is your biological sex?
    a. Man
    b. Woman

- With this question, more information is obtained about the characteristics of the health professional. It is not based on any of the hypotheses of the CP-FIT or REFLECT-52 theory. However, this question does connect to the theme of ‘health professional characteristics’ from the CP-FIT theory.
- To what extent do you think the content of this question is relevant to be included in the survey? (score 1 = not very relevant, score 9 = very relevant)

- Scale 1-9, 'do not rate' option.
- Justify your choice: (open)
- Suggestions for modification: (open)

4) Where is your practice located?
   a. Postcode: (open)
   b. Municipality: (open)

- With this question, more information is obtained about the characteristics of the practice in which the health professional works. It is not based on any of the hypotheses of the CP-FIT or REFLECT-52 theory. However, this question does connect to the theme of 'health professional characteristics' from the CP-FIT theory.
- To what extent do you think the content of this question is relevant to be included in the survey? (score 1 = not very relevant, score 9 = very relevant)
  - Scale 1-9, 'do not rate' option.
  - Justify your choice: (open)
  - Suggestions for modification: (open)

5) What type of practice do you work in?
   a. Solo practice
   b. Duo practice
   c. Group practice

- With this question, more information is obtained about the characteristics of the practice in which the health professional works. It connects to the theme 'resource' from the CP-FIT theory. This hypothesis states that teams and organizations have more capacity to process feedback.
- To what extent do you think the content of this question is relevant to be included in the survey? (score 1 = not very relevant, score 9 = very relevant)
  - Scale 1-9, 'do not rate' option.
  - Justify your choice: (open)
  - Suggestions for modification: (open)

6) Do you work in a multidisciplinary practice (presence of different disciplines such as paramedics or social worker)?
   a. Yes
   b. No

- With this question, more information is obtained about the characteristics of the practice in which the health professional works. It connects to the theme 'resource' from the CP-FIT theory. This hypothesis states that teams and organizations have more capacity to process feedback.
- To what extent do you think the content of this question is relevant to be included in the survey? (score 1 = not very relevant, score 9 = very relevant)
  - Scale 1-9, 'do not rate' option.

- Justify your choice: (open)
- Suggestions for modification: (open)

7) Does your practice employ a practice assistant and/or administrative assistant?
   a. Yes
   b. No

- With this question, more information is obtained about the characteristics of the practice in which the health professional works. It connects to the theme 'resource' from the CP-FIT theory. This hypothesis states that teams and organizations have more capacity to process feedback.
- To what extent do you think the content of this question is relevant to be included in the survey? (score 1 = not very relevant, score 9 = very relevant)
  - Scale 1-9, 'do not rate' option.
  - Justify your choice: (open)
  - Suggestions for modification: (open)

8) Does your practice currently employ a trainee general practitioner?
   a. Yes
   b. No

- With this question, more information is obtained about the characteristics of the practice in which the health professional works. It connects to the themes 'resource' and 'leadership support' from the CP-FIT theory. The 'resource' hypothesis states that teams and organizations have more capacity to process feedback. The 'leadership support' hypothesis states that when senior colleagues advocate for the feedback intervention or are committed to ensuring that this intervention is a success, they influence younger colleagues and create involvement.
- To what extent do you think the content of this question is relevant to be included in the survey? (score 1 = not very relevant, score 9 = very relevant)
  - Scale 1-9, 'do not rate' option.
  - Justify your choice: (open)
  - Suggestions for modification: (open)

9) How many in-person patient contacts (consultation and home visits) do you have on an average weekday?
   a. ≤10
   b. 11-20
   c. 21-30
   d. 31-40
   e. >40
- With this question, more information is obtained about the characteristics of the practice in which the health professional works. It connects to the theme 'competing priorities' from the CP-FIT theory. This hypothesis states that people who have significant other responsibilities are less able to handle and process feedback.

- To what extent do you think the content of this question is relevant to be included in the survey? (score 1 = not very relevant, score 9 = very relevant)
    - Scale 1-9, 'do not rate' option.
    - Justify your choice: (open)
    - Suggestions for modification: (open)

10) Which patient group do you consult with most frequently? Please rank from most frequent to least frequent?
    a. 0-17 years
    b. 18-64 years
    c. 65-84 years
    d. ≥85 years

- With this question, more information is obtained about the characteristics of the patient population in the practice in which the health professional works. It is not based on any of the hypotheses of the CP-FIT or REFLECT-52 theory. However, this question does connect to the theme ‘patient population’ from the CP-FIT theory.
- To what extent do you think the content of this question is relevant to be included in the survey? (score 1 = not very relevant, score 9 = very relevant)
    - Scale 1-9, 'do not rate' option.
    - Justify your choice: (open)
    - Suggestions for modification: (open)

11) Indicate the extent to which you agree with the following statement: I consider myself competent in using a computer.
    a. Totally agree
    b. Rather agree
    c. Neutral
    d. Rather disagree
    e. Totally disagree

- With this question, more information is obtained about the characteristics of the health professional. It corresponds to the theme ‘knowledge and skills’ from the CP-FIT theory. This hypothesis states that feedback is more effective if the recipients have greater knowledge (both clinical and technical).
- To what extent do you think the content of this question is relevant to be included in the survey? (score 1 = not very relevant, score 9 = very relevant)
    - Scale 1-9, 'do not rate' option.
    - Justify your choice: (open)
    - Suggestions for modification: (open)

12) Did you participate at least once in the Audit?
    a. Yes
    b. No

- This question is not based on a theme from the CP-FIT or REFLECT-52. However, it allows us to evaluate whether the participant used the A&F system.
- To what extent do you think the content of this question is relevant to be included in the survey? (score 1 = not very relevant, score 9 = very relevant)
    - Scale 1-9, 'do not rate' option.
    - Justify your choice: (open)
    - Suggestions for modification: (open)

If question 12 was answered with option 'a', go to question 14, if this question was answered with 'b', go to question 13.

13) Why did you not participate in the Audit? (open)

- This question is not based on a theme from the CP-FIT or REFLECT-52. However, it enables us to find out why people did not participate in the A&F system. The information obtained can be included when adjusting the A&F system.
- To what extent do you think the content of this question is relevant to be included in the survey? (score 1 = not very relevant, score 9 = very relevant)
    - Scale 1-9, 'do not rate' option.
    - Justify your choice: (open)
    - Suggestions for modification: (open)

*** Share practice data: Prioritizing Question 1-13. Select a top 5.**

Part 2 – This section questions the use of the Audit

Deel 2.1 Purpose of the survey

14) Please indicate the extent to which you agree with the following statements:

	Totally agree	Rather agree	Neutral	Rather agree	Totally disagree
14.1 The purpose of the Audit is clear to me.					
14.2 I find the purpose of the Audit meaningful.					
14.3 I find the purpose of the Audit relevant to the work I do today.					
14.4 I find that the use of the Audit is tailored to the specific needs of my practice.					
14.5 I find that the use of the Audit is consistent with my current way of working.					

14.6 I find that using the Audit positively affects my current way of working					

14.1

- With this question, more information is obtained about the purpose of the A&F system. This corresponds to the theme 'goal' from the CP-FIT theory and REFLECT-52.
- To what extent do you think the content of this question is relevant to be included in the survey? (score 1 = not very relevant, score 9 = very relevant)
  - Scale 1-9, 'do not rate' option.
  - Justify your choice: (open)
  - Suggestions for modification: (open)

14.2

- With this question, more information is obtained about the purpose of the A&F system. This corresponds to the theme ' importance ' from the CP-FIT theory. This hypothesis states that feedback is more effective when the intervention measures aspects of care that the recipient finds meaningful.
- To what extent do you think the content of this question is relevant to be included in the survey? (score 1 = not very relevant, score 9 = very relevant)
  - Scale 1-9, 'do not rate' option.
  - Justify your choice: (open)
  - Suggestions for modification: (open)

14.3

- With this question, more information is obtained about the purpose of the A&F system. This corresponds to the theme ' relevance ' from the CP-FIT theory. This hypothesis states that feedback is more effective when the intervention measures aspects of care that the recipient finds meaningfully relevant to the job he/she is doing.
- To what extent do you think the content of this question is relevant to be included in the survey? (score 1 = not very relevant, score 9 = very relevant)
  - Scale 1-9, 'do not rate' option.
  - Justify your choice: (open)
  - Suggestions for modification: (open)

14.4

- With this question, more information is obtained about the purpose of the A&F system. This corresponds to the theme ' adaptability ' from the CP-FIT theory. This hypothesis states that feedback is more successful when the intervention is tailored to the specific needs of the healthcare organization.
- To what extent do you think the content of this question is relevant to be included in the survey? (score 1 = not very relevant, score 9 = very relevant)
  - Scale 1-9, 'do not rate' option.
  - Justify your choice: (open)
  - Suggestions for modification: (open)

14.5

- With this question, more information is obtained about the purpose of the A&F system. This corresponds to the theme ' workflow fit ' from the CP-FIT theory. This hypothesis states that when the feedback intervention fits in with the existing ways of working of the healthcare organization, it takes less effort to implement the feedback.
- To what extent do you think the content of this question is relevant to be included in the survey? (score 1 = not very relevant, score 9 = very relevant)
    - Scale 1-9, 'do not rate' option.
    - Justify your choice: (open)
    - Suggestions for modification: (open)

14.6

- With this question, more information is obtained about the purpose of the A&F system. This corresponds to the theme ' feedback attitude ' from the CP-FIT theory. This hypothesis states that health professionals with positive views about the possible benefits of feedback are more likely to engage in feedback intervention.
- To what extent do you think the content of this question is relevant to be included in the survey? (score 1 = not very relevant, score 9 = very relevant)
    - Scale 1-9, 'do not rate' option.
    - Justify your choice: (open)
    - Suggestions for modification: (open)

*** Part purpose of survey: Prioritizing questions 14.1-14.6**

Part 2.2 Collection of data through the EMD

15) How satisfied are you with the way the data was collected? (Completing and submitting the e-form in the EMD)
    a. Very satisfied
    b. Rather satisfied
    c. Neutral
    d. Rather dissatisfied
    e. Very dissatisfied

- With this question, more information is obtained about the data collection through the A&F system. This corresponds to the themes 'automation' and 'conducted by recipients' from the CP-FIT theory. This hypothesis states that when the health professional has to collect data themself or manually, feedback intervention is less effective. Often a lack of time and/or knowledge is the root cause.
- To what extent do you think the content of this question is relevant to be included in the survey? (score 1 = not very relevant, score 9 = very relevant)
    - Scale 1-9, 'do not rate' option.
    - Justify your choice: (open)
    - Suggestions for modification: (open)

16) How could we improve the way we collect data? (open)

- This question is not based on the CP-FIT or REFLECT-52. However, it offers more insights into how we can improve the A&F system.

- To what extent do you think the content of this question is relevant to be included in the survey? (score 1 = not very relevant, score 9 = very relevant)
    - Scale 1-9, 'do not rate' option.
    - Justify your choice: (open)
    - Suggestions for modification: (open)

17) Indicate the extent to which you agree with the following statement: I feel that the data and results retrieved are an accurate representation of my practice.
    a. Totally agree
    b. Rather agree
    c. Neutral
    d. Rather disagree
    e. Totally disagree

- With this question, more information is obtained about the data collection through the A&F system. This corresponds to the theme ‘accuracy’ from the CP-FIT theory. This hypothesis states that feedback intervention is more effective when the receiver believes that the collected data is being presented truthfully.
- To what extent do you think the content of this question is relevant to be included in the survey? (score 1 = not very relevant, score 9 = very relevant)
    - Scale 1-9, 'do not rate' option.
    - Justify your choice: (open)
    - Suggestions for modification: (open)

18) How satisfied are you with the frequency of data retrieval?
    a. Very satisfied
    b. Rather satisfied
    c. Neutral
    d. Rather dissatisfied
    e. Very dissatisfied
    f. Totally disagree

- With this question, more information is obtained about the data collection through the A&F system. This corresponds to the themes ‘age of the data’ and ‘interval’ from the REFLECT-52 and ‘timeliness’ from de CP-FIT. This informs about the time intervals between the moments of data collection and the recency of the data used.
- To what extent do you think the content of this question is relevant to be included in the survey? (score 1 = not very relevant, score 9 = very relevant)
    - Scale 1-9, 'do not rate' option.
    - Justify your choice: (open)
    - Suggestions for modification: (open)

19) How frequently would you like to participate in the Audit?
    a. Daily
    b. Weekly
    c. Biweekly
    d. Monthly

    e. Quarterly
    f. Semi-annually
    g. Other: open

- With this question, more information is obtained about the data collection through the A&F system. This corresponds to the themes ‘cost’ and ‘workflow fit’ from the CP-FIT theory. This hypothesis states that interventions that are 'expensive' in terms of time, people or financial resources are generally less effective because they require more effort. However, if the intervention is more in line with the way in which the health professional wants to work, the intervention will be successful. That is why it is useful to query the desired frequency.
- To what extent do you think the content of this question is relevant to be included in the survey? (score 1 = not very relevant, score 9 = very relevant)
    - Scale 1-9, 'do not rate' option.
    - Justify your choice: (open)
    - Suggestions for modification: (open)

20) Indicate the extent to which you agree with the following statement: I find participation in the Audit to be little labor intensive.
    a. Totally agree
    b. Rather agree
    c. Neutral
    d. Rather disagree
    e. Totally disagree

- With this question, more information is obtained about the data collection through the A&F system. This corresponds to the theme ‘cost’ from the CP-FIT theory. This hypothesis states that interventions that are 'expensive' in terms of time, people or financial resources are generally less effective because they require more effort.
- To what extent do you think the content of this question is relevant to be included in the survey? (score 1 = not very relevant, score 9 = very relevant)
    - Scale 1-9, 'do not rate' option.
    - Justify your choice: (open)
    - Suggestions for modification: (open)

*** Part collection of data through the EMD: Prioritization question 15-20**

Part 2.3 Get feedback

21) How frequently did you view the feedback?
    a. Never
    b. Weekly
    c. Monthly
    d. Quarterly
    e. Semi-annually
    f. Other: open

- With this question, more information is obtained about the feedback received through the A&F system. This corresponds to the theme ‘engagement assessment’ from the REFLECT-52. This question assesses whether the healthcare professional participated in the A&F system.
- To what extent do you think the content of this question is relevant to be included in the survey? (score 1 = not very relevant, score 9 = very relevant)
    - Scale 1-9, 'do not rate' option.
    - Justify your choice: (open)
    - Suggestions for modification: (open)

If you indicated answer 'a' on question 21, go to question 22. If you chose 'b, c, d, e or f,' go to question 23.

22) Why did you never look at the feedback? (open)

- With this question, more information is obtained about the feedback received through the A&F system. This corresponds to the theme ‘engagement assessment’ from the REFLECT-52. This question assesses whether the healthcare professional participated in the A&F system.
- To what extent do you think the content of this question is relevant to be included in the survey? (score 1 = not very relevant, score 9 = very relevant)
    - Scale 1-9, 'do not rate' option.
    - Justify your choice: (open)
    - Suggestions for modification: (open)

23) How satisfied are you…

	Totally satisfied	Rather satisfied	Neutral	Rather satisfied	Totally satisfied
23.1 about how feedback is given (in general)?					
23.2 about the frequency of the feedback?					
23.3 about getting feedback per practice, as opposed to being able to get feedback per physician?					
23.4 about the way the performance level of the practice is displayed?					
23.5 about the number of charts and tables that can be accessed?					
23.6 about the content of the feedback?					
23.7 about the possibility to compare performance level of the practice with others (province/ primary care area/...)? (benchmarking)					

23.8 about the number of levels (county/first-line zone/...) available for comparison?					

23.1

- With this question, more information is obtained about the characteristics of the received feedback. It is not based on any of the hypotheses of the CP-FIT or REFLECT-52 theory.
- To what extent do you think the content of this question is relevant to be included in the survey? (score 1 = not very relevant, score 9 = very relevant)
  - Scale 1-9, 'do not rate' option.
  - Justify your choice: (open)
  - Suggestions for modification: (open)

23.2

- With this question, more information is obtained about the feedback received through the A&F system. This corresponds to the theme ‘number of feedback reports’ from REFLECT-52.
- To what extent do you think the content of this question is relevant to be included in the survey? (score 1 = not very relevant, score 9 = very relevant)
  - Scale 1-9, 'do not rate' option.
  - Justify your choice: (open)
  - Suggestions for modification: (open)

23.3

- With this question, more information is obtained about the feedback received through the A&F system. This corresponds to the themes ‘specificity’, ‘performance level’ from CP-FIT and ‘individual performance’, ‘group performance’ and ‘group level’ from REFLECT-52. This hypothesis states that the intervention is more effective when the feedback provided is reported to the individual health professional.
- To what extent do you think the content of this question is relevant to be included in the survey? (score 1 = not very relevant, score 9 = very relevant)
  - Scale 1-9, 'do not rate' option.
  - Justify your choice: (open)
  - Suggestions for modification: (open)

23.4

- With this question, more information is obtained about the feedback received through the A&F system. This corresponds to the themes ‘usability’ from de CP-FIT and ‘format’ and ‘graphical elements’ from REFLECT-52. This hypothesis states that the feedback is more effective when user-friendly designs are chosen.
- To what extent do you think the content of this question is relevant to be included in the survey? (score 1 = not very relevant, score 9 = very relevant)
  - Scale 1-9, 'do not rate' option.
  - Justify your choice: (open)
  - Suggestions for modification: (open)

23.5

- With this question, more information is obtained about the feedback received through the A&F system. This corresponds to the theme ‘number of graphs or tables’ from REFLECT-52.
- To what extent do you think the content of this question is relevant to be included in the survey? (score 1 = not very relevant, score 9 = very relevant)
  - Scale 1-9, 'do not rate' option.
  - Justify your choice: (open)
  - Suggestions for modification: (open)

23.6

- With this question, more information is obtained about the feedback received through the A&F system. This corresponds to the theme ‘outcome type’ from REFLECT-52 and describes the content of the feedback.
- To what extent do you think the content of this question is relevant to be included in the survey? (score 1 = not very relevant, score 9 = very relevant)
  - Scale 1-9, 'do not rate' option.
  - Justify your choice: (open)
  - Suggestions for modification: (open)

23.7

- With this question, more information is obtained about the feedback received through the A&F system. This corresponds to the themes ‘previous performance’, ‘discrepancy’ and ‘comparator’ from REFLECT-52 and ‘benchmarking’ from CP-FIT. This hypothesis states that benchmarking can motivate health professionals to improve their performance.
- To what extent do you think the content of this question is relevant to be included in the survey? (score 1 = not very relevant, score 9 = very relevant)
  - Scale 1-9, 'do not rate' option.
  - Justify your choice: (open)
  - Suggestions for modification: (open)

23.8

- With this question, more information is obtained about the feedback received through the A&F system. This corresponds to the themes ‘previous performance’, ‘discrepancy’ and ‘comparator’ from REFLECT-52 and ‘benchmarking’ from CP-FIT. This hypothesis states that benchmarking can motivate health professionals to improve their performance.
- To what extent do you think the content of this question is relevant to be included in the survey? (score 1 = not very relevant, score 9 = very relevant)
  - Scale 1-9, 'do not rate' option.
  - Justify your choice: (open)
  - Suggestions for modification: (open)

***Part 1: getting feedback. Prioritizing questions 23.1-23.8**

24) Please indicate the extent to which you agree with the following statements:

	Totally agree	Rather agree	Neutral	Rather agree	Totally disagree
24.1 I would rather get feedback about the individual patient.					
24.2 I find the feedback given relevant to achieving a better level of performance.					
24.3 I find it useful that the current level of performance is shown in relation to the previous level of performance (longitudinal view of performance level).					
24.4 I find the way a feedback report can be viewed user-friendly.					

24.1

- With this question, more information is obtained about the feedback received through the A&F system. This corresponds to the themes ‘individual data’ and ‘aggregated data’ from REFLECT-52 and ‘patient lists’ from CP-FIT. This hypothesis states that displaying patient data used to assess the clinical performance of the health professional, facilitates the feedback mechanism by enabling recipients to understand how suboptimal care arose.
- To what extent do you think the content of this question is relevant to be included in the survey? (score 1 = not very relevant, score 9 = very relevant)
  - Scale 1-9, 'do not rate' option.
  - Justify your choice: (open)
  - Suggestions for modification: (open)

24.2

- With this question, more information is obtained about the feedback received through the A&F system. This corresponds to the theme ‘link to feedback’ from REFLECT-52 and and checks whether the feedback is aimed at the goals of the A&F system.
- To what extent do you think the content of this question is relevant to be included in the survey? (score 1 = not very relevant, score 9 = very relevant)
  - Scale 1-9, 'do not rate' option.
  - Justify your choice: (open)
  - Suggestions for modification: (open)

24.3

- With this question, more information is obtained about the feedback received through the A&F system. This corresponds to the themes ‘previous performance’ from REFLECT-52 and ‘trend’ from CP-FIT. This hypothesis states that a longitudinal representation of

the results facilitates understanding of feedback, because the receiver can place their own results in a historical context.
- To what extent do you think the content of this question is relevant to be included in the survey? (score 1 = not very relevant, score 9 = very relevant)
  - Scale 1-9, 'do not rate' option.
  - Justify your choice: (open)
  - Suggestions for modification: (open)

24.4

- With this question, more information is obtained about the feedback received through the A&F system. This corresponds to the theme ‘usability’ from CP-FIT. This hypothesis states that feedback presented with a user-friendly design is more effective.
- To what extent do you think the content of this question is relevant to be included in the survey? (score 1 = not very relevant, score 9 = very relevant)
  - Scale 1-9, 'do not rate' option.
  - Justify your choice: (open)
  - Suggestions for modification: (open)

***Part 2: getting feedback. Prioritizing questions 24.1-24.4**

25) Did you set yourself a goal regarding the level of performance to be achieved based on the feedback given?
    a. Yes
    b. No

- With this question, more information is obtained about the feedback received through the A&F system. This corresponds to the theme ‘goal’ from REFLECT-52 and checks whether the health professional set their own goals.
- To what extent do you think the content of this question is relevant to be included in the survey? (score 1 = not very relevant, score 9 = very relevant)
  - Scale 1-9, 'do not rate' option.
  - Justify your choice: (open)
  - Suggestions for modification: (open)

26) Optional: Why did you/didn’t you set a goal? (open)
- With this question, more information is obtained about the feedback received through the A&F system. This corresponds to the theme ‘goal’ from REFLECT-52 and checks whether the health professional set their own goals.
- To what extent do you think the content of this question is relevant to be included in the survey? (score 1 = not very relevant, score 9 = very relevant)
  - Scale 1-9, 'do not rate' option.
  - Justify your choice: (open)
  - Suggestions for modification: (open)

27) Please indicate the extent to which you agree with the following statements:

	Totally agree	Rather agree	Neutral	Rather agree	Totally disagree
27.1 I find the effort I have to put into reviewing the feedback negligible.					
27.2 I am satisfied with the amount of feedback given.					
27.3 The feedback should contain written advice that aims to improve my performance level and is easy to implement.					
27.4 If I received the feedback directly in my EMD (push system), I would view it more frequently.					
27.5 The feedback provided aims to support me in improving the performance level of my practice.					
27.6 It is important to me that the feedback provided on my participation is not visible to third parties.					
27.7 I am convinced that the feedback provided benefits me/is beneficial to me.					
27.8 I find it important that the feedback provided is delivered by a professional organization.					
27.9 The feedback given has led to changes in my medical practice.					

27.1

- With this question, more information is obtained about the feedback received through the A&F system. This corresponds to the theme ‘cost’ from CP-FIT. This hypothesis states that the interventions that are ‘expensive’ in terms of time, people or financial resources are generally less effective because they require more effort.
- To what extent do you think the content of this question is relevant to be included in the survey? (score 1 = not very relevant, score 9 = very relevant)
    - Scale 1-9, 'do not rate' option.
    - Justify your choice: (open)
    - Suggestions for modification: (open)

27.2

- With this question, more information is obtained about the feedback received through the A&F system. This corresponds to the theme ‘number of pages’ from REFLECT-52 and and checks how long the feedback report is.

- To what extent do you think the content of this question is relevant to be included in the survey? (score 1 = not very relevant, score 9 = very relevant)
    - Scale 1-9, 'do not rate' option.
    - Justify your choice: (open)
    - Suggestions for modification: (open)

27.3

- With this question, more information is obtained about the feedback received through the A&F system. This corresponds to the themes ‘action planning’ from CP-FIT and ‘actionable messages’ from REFLECT-52. This hypothesis states that short, actionable advice that enables the health professional to improve its outcome should be given when providing feedback.
- To what extent do you think the content of this question is relevant to be included in the survey? (score 1 = not very relevant, score 9 = very relevant)
    - Scale 1-9, 'do not rate' option.
    - Justify your choice: (open)
    - Suggestions for modification: (open)

27.4

- With this question, more information is obtained about the feedback received through the A&F system. This corresponds to the theme ‘active delivery’ from CP-FIT. This hypothesis states that feedback sent to the receiver through a push system is more effective than through a pull system.
- To what extent do you think the content of this question is relevant to be included in the survey? (score 1 = not very relevant, score 9 = very relevant)
    - Scale 1-9, 'do not rate' option.
    - Justify your choice: (open)
    - Suggestions for modification: (open)

27.5

- With this question, more information is obtained about the feedback received through the A&F system. This corresponds to the themes ‘function’ from CP-FIT and ‘defensive reactions’ from REFLECT-52. This hypothesis states that feedback is more effective when the receiver believes it is intended to support change rather than punish suboptimal performance.
- To what extent do you think the content of this question is relevant to be included in the survey? (score 1 = not very relevant, score 9 = very relevant)
    - Scale 1-9, 'do not rate' option.
    - Justify your choice: (open)
    - Suggestions for modification: (open)

27.6

- With this question, more information is obtained about the feedback received through the A&F system. This corresponds to the theme ‘delivery to a group’ from CP-FIT. This hypothesis states that when the feedback is also publicly available, it can elicit negative responses, but with little impact on clinical performance.
- To what extent do you think the content of this question is relevant to be included in the survey? (score 1 = not very relevant, score 9 = very relevant)

- Scale 1-9, 'do not rate' option.
- Justify your choice: (open)
- Suggestions for modification: (open)

27.7

- With this question, more information is obtained about the feedback received through the A&F system. This corresponds to the theme ‘feedback attitude’ from CP-FIT. This hypothesis states that health professionals with positive views about the possible benefits of feedback are more likely to engage in the feedback intervention
- To what extent do you think the content of this question is relevant to be included in the survey? (score 1 = not very relevant, score 9 = very relevant)
  - Scale 1-9, 'do not rate' option.
  - Justify your choice: (open)
  - Suggestions for modification: (open)

27.8

- With this question, more information is obtained about the feedback received through the A&F system. This corresponds to the themes ‘source knowledge and skills’ from CP-FIT and ‘Feedback delivery-organization’ from REFLECT-52. This hypothesis states that feedback is more effective when the recipient believes it is being delivered by a person or organization that is considered to have an appropriate level of knowledge.
- To what extent do you think the content of this question is relevant to be included in the survey? (score 1 = not very relevant, score 9 = very relevant)
  - Scale 1-9, 'do not rate' option.
  - Justify your choice: (open)
  - Suggestions for modification: (open)

27.9

- With this question, more information is obtained about the feedback received through the A&F system. This corresponds to the theme ‘behavioral response’ from CP-FIT. This question examines whether the feedback has brought about a change in behavior.
- To what extent do you think the content of this question is relevant to be included in the survey? (score 1 = not very relevant, score 9 = very relevant)
  - Scale 1-9, 'do not rate' option.
  - Justify your choice: (open)
  - Suggestions for modification: (open)

28) Why has the feedback given led to changes in your medical practice or not? (open)

- With this question, more information is obtained about the feedback received through the A&F system. This corresponds to the theme ‘barriers assessment’ from CP-FIT. This question examines the barriers that prevented the health professional from changing their behavior.
- To what extent do you think the content of this question is relevant to be included in the survey? (score 1 = not very relevant, score 9 = very relevant)
  - Scale 1-9, 'do not rate' option.
  - Justify your choice: (open)

- Suggestions for modification: (open)

***Part 3: getting feedback. Prioritizing questions 27.1-28**

***Part 4: getting feedback. Prioritizing questions 21,22,25,26**

Part 2.4 Co-interventions

29) Please indicate the extent to which you agree with the following statement: A discussion with other GPs regarding the feedback received seems a useful addition in order to arrive at changes in my medical practice.
    a. Totally agree
    b. Rather agree
    c. Neutralfrom
    d. Rather disagree
    e. Totally disagree

- With this question, more information is obtained about the feedback received through the A&F system. This corresponds to the themes ‘peer discussion’ from de CP-FIT and ‘social context’ from REFLECT-52. This hypothesis states that feedback is more effective when the receiver has the opportunity to discuss the feedback received with others.
- To what extent do you think the content of this question is relevant to be included in the survey? (score 1 = not very relevant, score 9 = very relevant)
    - Scale 1-9, 'do not rate' option.
    - Justify your choice: (open)
    - Suggestions for modification: (open)

30) How satisfied are you regarding education on using the Audit and getting feedback (webinar, film clip)?
    a. Very satisfied
    b. Rather satisfied
    c. Neutral
    d. Rather dissatisfied
    e. Very dissatisfied
    f. I haven’t looked into it

- With this question, more information is obtained about the education through the A&F system. This corresponds to the theme ‘training’ from CP-FIT. This hypothesis states that feedback is more effective when education is provided on how to obtain the feedback.
- To what extent do you think the content of this question is relevant to be included in the survey? (score 1 = not very relevant, score 9 = very relevant)
    - Scale 1-9, 'do not rate' option.
    - Justify your choice: (open)
    - Suggestions for modification: (open)

31) How satisfied are you with the way questions and problems were resolved via e-mail?
    a. Very satisfied

b. Rather satisfied
c. Neutral
d. Rather dissatisfied
e. Very dissatisfied

- With this question, more information is obtained about the help received in using the A&F system. This corresponds to the themes ‘problem solving’ and ‘support’ from CP-FIT. These hypotheses state that feedback is more effective when the recipient is supported in obtaining feedback and solving problems.
- To what extent do you think the content of this question is relevant to be included in the survey? (score 1 = not very relevant, score 9 = very relevant)
  - Scale 1-9, 'do not rate' option.
  - Justify your choice: (open)
  - Suggestions for modification: (open)

32) How satisfied are you with the weekly reminder to participate sent to you by email?
a. Very satisfied
b. Rather satisfied
c. Neutral
d. Rather dissatisfied
e. Very dissatisfied

- With this question, more information is obtained about the guidance on using the A&F system. This corresponds to the theme ‘support’ from CP-FIT. This hypothesis states that feedback is more effective when the receiver is supported in obtaining feedback.
- To what extent do you think the content of this question is relevant to be included in the survey? (score 1 = not very relevant, score 9 = very relevant)
  - Scale 1-9, 'do not rate' option.
  - Justify your choice: (open)
  - Suggestions for modification: (open)

33) Indicate the extent to which you agree with the following statement: If I were financially compensated for my participation, I would participate more frequently.
a. Totally agree
b. Rather agree
c. Neutral
d. Rather disagree
e. Totally disagree

- With this question, more information is obtained about the A&F system. This corresponds to the theme ‘financial reward (cost)’ from CP-FIT. This hypothesis states that a financial reward may negatively impact feedback success by counteracting the recipient's motivation and sense of professionalism.
- To what extent do you think the content of this question is relevant to be included in the survey? (score 1 = not very relevant, score 9 = very relevant)
  - Scale 1-9, 'do not rate' option.
  - Justify your choice: (open)
  - Suggestions for modification: (open)

34) Is there anything else you would like to mention? (open)

- This question is not based on the CP-FIT or REFLECT-52. However, it offers more insights into how we can improve the A&F system.
- To what extent do you think the content of this question is relevant to be included in the survey? (score 1 = not very relevant, score 9 = very relevant)
    - Scale 1-9, 'do not rate' option.
    - Justify your choice: (open)
    - Suggestions for modification: (open)

***Part co-intervention. Prioritize question 29-34**
